# Struma ovarii associated with pseudo-Meigs' syndrome and elevated serum CA 125: a case report and review of the literature

**DOI:** 10.1186/1757-2215-3-18

**Published:** 2010-07-29

**Authors:** Wei Jiang, Xin Lu, Zhi Ling Zhu, Xi Shi Liu, Cong Jian Xu

**Affiliations:** 1 Department of Gynecology, Obstetrics and Gynecology Hospital, Fudan University, Shanghai, P.R. China

## Abstract

The association of pseudo-Meigs' syndrome, elevation of CA 125 to the struma ovarii is a rare condition. So far only nine cases have been reported in English literature through MEDLINE search. Here we report a 46-year-old case of the struma ovarii, presented with ascites, hydrothorax, right ovarian mass and elevated serum CA 125 level. These findings were misdiagnosed for an ovarian malignancy at the first impression. Immediate resolution of the ascites, hydrothorax and normalization of the serum CA 125 level were followed by ovarian mass removal. Struma ovarii could be a rare cause of ascites, hydrothorax, ovarian mass and elevated CA 125. This rare condition should be considered in the differential diagnosis in patents with ascites and pleural effusions but with negative cytology.

## Background

Struma ovarii is a rare ovarian neoplasm derived from germ cells in a mature teratoma. This tumor is generally benign, although malignant transformation has been reported [1]. The preoperative diagnosis is generally difficult. Thyroid hormones may be produced and in a few cases asymptomatic women may develop definitive clinical hypothyroidism after resection of struma ovarii. We here report an unusual case of a 46-year-old woman presented with ascites, right ovarian mass, and elevated CA 125 level, which was suspicious for an ovarian malignancy and underwent a total hysterectomy and bilateral salpingo-oophorectomy. The pathologic diagnosis was struma ovarii, a specialized ovarian teratoma composed predominantly of mature thyroid tissue. The postoperative period was uneventful and her thyroid function was normal. We had reviewed the related literatures in this report as well.

## Case presentation

The present case is a 46-year-old, female, gravida 1, para 1, who was admitted to a local hospital, complaining of fatigue, anorexia, and abdominal swelling. Her medical history included nothing special. Physical examination revealed a palpable mass in the lower abdomen. A thoracoabdominal CT scan showed marked pleural effusion and a heterogeneous mass, large ascites with many nodosity images in the pelvic wall and considered as malignant tumor of ovary.

She was then transferred to our hospital for further treatment in September, 2009. The patient's serum CA 125 level was 1230.9 U/mL, while CEA (2.6 ng/ml), AFP (14.2 ng/ml), CA 199 (14.8 U/ml), and CA 153 (7.8 U/ml) levels were within the normal range. Abdominal ultrasonography showed a heterogeneous, multiloculated mass, with a moderate amount of ascites, and subsequent transvaginal ultrasonography revealed a large complex pelvic mass, 16 cm largest dimension, of probable adnexal origin with low blood resistance flow within the tumor. The uterus was normal in size. Abdominal paracentesis yielded 2 liters of yellow serous fluid consistent with an exudative process. Microscopy and cytology revealed only reactive mesothelial cells without malignant cells.

The patient was arranged for an exploratory laparotomy. Six liters of straw-colored ascites was evacuated. The uterus was in normal size and the left ovary measured 3 × 2 × 2 cm with a normal appearance. A 20 × 18 × 15 cm complex, multicystic mass, without evidence of external excrescences, had replaced the right ovary. There was no evidence of intraperitoneal (ie. omenta, the surface of convolutions, appendix, liver, etc) spread of disease or retroperitoneal adenopathy. And right salpingo-oophorectomy was performed. A frozen section of the right ovarian mass was interpreted as struma ovarii. As strongly insisted by the patient and her family member, a subsequent hysterectomy and left salpingo-oophorectomy were performed according to the informed consent.

**Figure 1:**
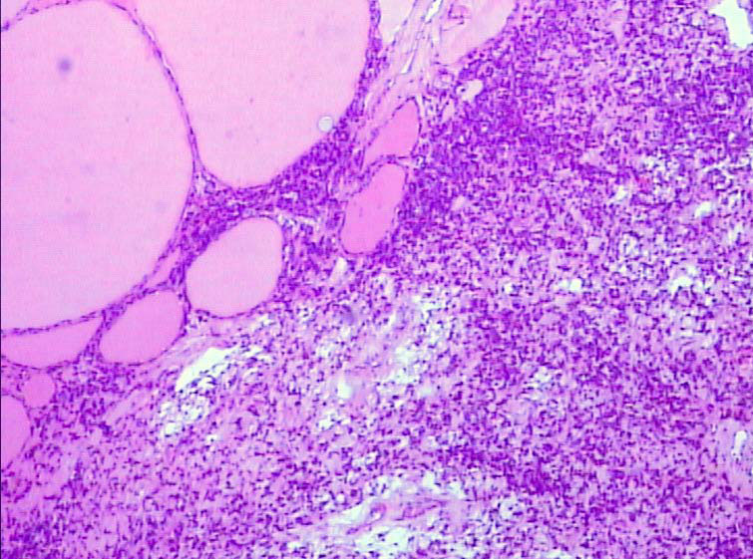
**Microscopic appearance of the right ovary showing thyroid follicles of varying sizes**. (H & E, 100×).

Post operative thyroid function test including serum levels of TT3 (1.78 nmo1/L), TT4 (82.5 nmo1/L), FT3 (8.2 pmol/L), FT4 (30.5 pmol/L) and TSH (2.3 mU/ml) were performed on day two, which were within normal limits. The level of CA 125 was decreased to 817 U/mL. The final pathology revealed right struma ovarii with benign thyroid tissue confined to the ovary (Figure 1). The uterus, left ovary, fallopian tube were histologically unremarkable and the cytologic evaluation of the ascitic fluid showed no evidence of malignant cells.

The patient recovered uneventfully and was discharged home on the ninth postoperative day with a CA 125 level of 485 U/mL. Following up three months after her surgery, she had no evidence of ascites and the serum levels of CA 125 was in normal range, she was symptomatically much improved from her preoperative condition and received hormone replacement therapy.

## Discussion

Mature cystic teratomas account for approximately 20% of all ovarian tumors. Of these, approximately 15% contain normal thyroid tissue. Struma ovarii is a monodermal variant of ovarian teratoma, which predominantly contains thyroid tissue (greater than 50%) and was first described by Von Klden in 1895 and Gottschalk in 1899 [2]. It constitutes about 2.7% of ovarian teratomas. It is usually a benign condition although occasionally, malignant transformation is observed. Preoperative clinical diagnosis of struma ovarii, however, is very difficult.

Despite containing thyroid tissue, only 5% of struma ovarii have features of hyperthyroidism [3]. Ascites has been reported in one-third of cases [2]. However, uncommon is the association of ascites and hydrothorax with this tumor [2]. Meigs first described the syndrome consisting of ovarian fibroma/thecoma, with ascites and hydrothorax, characterized by the resolution of symptoms with removal of the benign tumor [2]. Meigs' syndrome proposed to benign and solid tumors with the gross appearance of a fibroma (fibroma, thecoma, granulosa cell tumor), accompanied by ascites and hydrothorax. While similar clinic manifestations presented in other conditions was termed as pseudo-Meigs syndrome. The ascitic and pleural fluids in Meigs' and pseudo-Meigs' syndrome are usually serous, but may be serosanguinous. The origin of the effusions remains obscure, although some mechanisms have been suggested such as active fluid secretion by the tumor or peritoneum, venous and/or lymphatic obstruction, low serum protein and inflammatory products [4].

In the literature, very few reports have been published on struma ovarii associated to ascites and elevated CA125 [5-8]. In both cases, patients presented with ascites but without pleural effusions. A MEDLINE search of the English language literature provides only nine case report describing struma ovarii presenting as pseudo-Meigs' syndrome with an elevated CA 125 level can initially suggest ovarian carcinoma [9-16]. (Table 1) We describe an additional case to the tenth reported in the literature with struma ovarii associated with pseudo-Meigs syndrome and elevated CA 125, which shows analogies with the ones reported in the literature. It differs in some important respects. Firstly the patient's age, this is much younger than that when the majority of these tumors occur i.e. in the fifties. Secondly, the patient underwent a wide resection operation because of the strong desire of both the patient and her husband and received a hormone replacement therapy subsequently.

**Table 1:** Struma ovarii associated with Pseudo-Meigs' syndrome and elevated CA125 level: reported cases

Author	No. of patients	Age (years)	Clinical symptoms	CA 125 (U/mL)	Treatments	Prognosis & follow up time
Bethune M *et al. *_(9)_	1	62	Acute hydrothoraces, dyspnea and abdominal swelling	1570	Total hysterectomy and bilateral Salpingo-oophorectomy	Well, 5 months
Long CY *et a.l *^(10)^	2	53 78	Both with abdominal swelling, pain, or dyspnea	233 335	Both with total hysterectomy and bilateral Salpingo-oophorectomy	Well, 10 months Well, 6 months
Huh JJ *et al. *^(11)^	1	65	Abdominal distension, dyspnea	402	Total hysterectomy and bilateral Salpingo-oophorectomy and appendectomy and omental biopsy	Well, 4 months
Loizzi V *et al. *^(12)^	1	65	Dyspnea, diffuse abdominal pain	161	Not included	Well, 2 months
Mitrou S *et al*.^(13)^	1	58	Large pelvic mass, ascites	1028	Total hysterectomy and bilateral Salpingo-oophorectomy	Well, 12 months
Paladini D, *et al*^(14)^	1	42	Ascites, fever, diarrhea, vomiting and significant weight loss.	2548	Right Salpingo-oophorectomy	Well, 6 months
Obeidat BR, *et al*^(15)^	1	67	Dyspnea, abdominal swelling, pelvic mass	176	Total hysterectomy and bilateral Salpingo-oophorectomy	Well, 6 months
Rana V, *et al*^(16)^	1	70	Progressive ascites, bilateral pleural effusion	284	total abdominal hysterectomy with bilateral Salphingo- opherectomy and partial omentectomy	Well, 3 months
Present case	1	46	Abdominal swelling, fatigue, weight loss	1230.9	Total hysterectomy and bilateral Salpingo-oophorectomy	Well, 3 months

The elevation of CA 125 may have been secondary to the presence of ascites; however, its level was much higher than that typically seen with ascites of benign origin. An ovarian mass with ascites and elevated serum CA 125 level in a woman generally suggest a malignancy process. So the present case with the clinic findings of ascites, hydrothorax, markedly elevated serum CA 125 and a large complex pelvic mass in a woman strongly suggest pelvic malignancy before operation. But complete remission of the ascites, hydrothorax, and CA125 was obtained after surgery without any adjuvant therapy.

## Conclusion

This report emphasizes that there are benign gynecological conditions might show clinical, ultrasonographic and biochemical signs suggestive of malignancy. They rarely should be considered as the benign diseases in the differential diagnosis when the patients presented with ascites, elevated serum CA 125 and pleural effusions, but with negative cytologic examination.

## List of abbreviations

CT: computed tomography; TSH: thyroid stimulating hormone; CEA: carcinoembryonic antigen; AFP: alpha-fetoprotein; T3: triiodothyronine; T4: thyroxine; TT3: total T3; TT4: total T4; FT3: free T3; FT4: free T4.

## Competing interests

The authors declare that they have no competing interests.

## Authors' contributions

WJ drafted the manuscript. XL, ZJZ, CJX, XSL are involved in design, acquisition, interpretation and manuscript preparation. All authors had read and approved the final manuscript.

## Authors' information

WJ, XL, ZLZ, CJX, XSL: Department of Gynecology, Obstetrics and Gynecology Hospital, Fudan University, Shanghai, P. R. China.

## Consent

Written informed consent was obtained from the patient for publication of this case report and any accompanying images. A copy of the written consent is available for review by the Editor-in-Chief of this journal.
